# Abstract of Meteorological Observations for November, 1854, Made at Philadelphia, Pa.

**Published:** 1855-01

**Authors:** James A. Kirkpatrick


*Abstract of Meteorological Observations for November,* 1854, *made at Philadelphia, Pa. Latitude* 39° 57′ 28″ *N., Longitude* 75° 10′ 40″ *W. from Greenwich.* By PROF. JAMES A. KIRKPATRICK.

| 1854. Nov. | BAROMETER. Daily Mean | BAROMETER. Mean Daily Range. | THERMOM. Daily Mean | THERMOM. Mean Daily Range | Dew Point 2 P. M. | Rel. Humid. 2 P. M. | Rain. | Prevailing Winds. | General Remarks. |
|---|---|---|---|---|---|---|---|---|---|
| | Inches. | Inches. | Deg. | Deg. | Deg. | Hunds. | Inch. | Points. | |
| 1 | 29.767 | .047 | 60.2 | 5.8 | 45.7 | .52 | | NW. | M. cl'r; aft. & ev. cl'y. *Th. hig'st* 69°. |
| 2 | 30.030 | .263 | 52.5 | 7.7 | 38.3 | .52 | | W. | Clear. |
| 3 | 30.033 | .034 | 56.7 | 5.2 | 41.7 | .49 | 0.150 | (Var.) | M. clear; aft. and ev. cl'y; night rain. |
| 4 | 30.239 | .206 | 43.3 | 10.0 | 30.7 | .48 | | N. | M. cloudy; aft. and ev. clear. |
| 5 | 30.357 | .115 | 32.7 | 10.7 | 26.7 | .60 | | N. | Clear. *First Ice. Bar. highest* 30.369; *Therm. lowest* 27°. |
| 6 | 30.023 | .334 | 40.3 | 7.7 | 30.7 | .48 | | SW. | Cloudy. |
| 7 | 29.549 | .475 | 48.7 | 8.3 | 40.0 | .62 | 0.023 | NW. | M. rain; aft. cloudy; ev. clear. |
| 8 | 29.829 | .280 | 45.7 | 3.0 | 32.7 | .50 | | (Var.) | Clear. |
| 9 | 30.110 | .314 | 46.3 | 4.0 | 37.7 | .56 | | SW. | Clear. |
| 10 | 30.074 | .080 | 52.7 | 7.0 | 50.3 | .72 | | S. | Cloudy. |
| 11 | 29.817 | .258 | 63.0 | 10.3 | 62.7 | .94 | 0.483 | SE. | M. and aft. rain; ev. clouoy. |
| 12 | 29.857 | .071 | 58.3 | 4.7 | 57.7 | .94 | 1.619 | E.NE. | Rain all day. |
| 13 | 29.724 | .141 | 58.3 | 9.7 | 49.0 | .67 | | (Var.) | Cloudy. |
| 14 | 29.871 | .147 | 40.2 | 18.2 | 30.0 | .52 | | NW. | Clear. |
| 15 | 29.541 | .330 | 42.5 | 2.0 | 38.0 | .77 | | (Var.) | Cloudy. |
| 16 | 29.587 | .116 | 40.7 | 1.8 | 24.3 | .36 | | NW. | M. clear; aft. and ev. cloudy. |
| 17 | 29.527 | .123 | 44.8 | 3.2 | 36.0 | .58 | | SW. | M. clear; aft. and ev. cloudy. |
| 18 | 29.582 | .135 | 46.2 | 5.0 | 35.3 | .55 | | NW. | Cloudy. |
| 19 | 29.728 | .143 | 39.3 | 6.8 | 30.0 | .52 | | NW. | Clear. |
| 20 | 29.862 | .134 | 38.7 | 1.3 | 29.3 | .57 | | W. | M. clear; aft. and ev. cloudy. |
| 21 | 29.980 | .118 | 42.7 | 3.7 | 31.7 | .49 | | SW. | Cloudy. |
| 22 | 29.824 | .122 | 46.8 | 4.2 | 41.0 | .78 | 0.294 | (Var.) | M. rain; aft. cloudy; ev. clear. |
| 23 | 29.898 | .086 | 45.8 | 3.3 | 41.0 | .69 | | SW. | Clear. |
| 24 | 29.462 | .436 | 54.0 | 8.2 | 57.3 | .97 | 0.781 | SE. | Rain all day. |
| 25 | 29.332 | .212 | 52.0 | 5.8 | 41.0 | .62 | 0.110 | SW. | Cloudy; ev. rain; *Bar. lowest* 29.257. |
| 26 | 29.663 | .331 | 42.7 | 9.3 | 27.3 | .40 | | SW. | M. clear; aft. and ev. cloudy. |
| 27 | 29.940 | .277 | 37.8 | 4.8 | 29.3 | .57 | | SW. | Clear. |
| 28 | 30 027 | .087 | 36.7 | 1.2 | 31.7 | .65 | | SW. | Clear. |
| 29 | 29.870 | .158 | 36.2 | 0.5 | 28.3 | .56 | | W.NW. | Clear. |
| 30 | 29.838 | .082 | 38.0 | 3.8 | 27.0 | .49 | | NW. | M. cl'y; slight snow; aft. & ev. clear. |
| Means for Nov. 1854 | 29.831 | .188 | 46.1 | 5.9 | 37.4 | .60 | 3.460 | S. 85° W. 61—100. | |
| Means for Nov. 1853 | 30.157 | | 47.1 | | 42.2 | | 2.320 | | |
| Means for Nov. 1852 | 29.919 | | 42.2 | | 35.2 | | 6.050 | | |
| Means for Nov. 3yrs | 29.969 | | 45.1 | | 38.3 | | 3.943 | N. 71° W. 45—100. | |

The extreme range of the barometer during the month was 1.112 inches, and of the thermometer 42°.

The observations are taken at 7, A.M., 2 and 9, P.M., daily. The *daily mean height* of the Barometer and Thermometer is found by taking the mean of the three observations; and the *monthly mean* determined by adding together the daily means and dividing by the number of days in the month. The *mean daily range* is found by calculating the mean of the differences between the height of the mercury on any particular day and the day immediately preceding, at the corresponding hours. The *Dew Point* and *Relative Humidity* are calculated from the difference between the wet and dry-bulb thermometers at 2, P.M. The *Resultant of the Winds for the Month* is found by Schow's method. The Barometrical Observations are all corrected for temperature by being reduced to 32° Fahrenheit.

